# Metabolic Changes Following Perinatal Asphyxia: Role of Astrocytes and Their Interaction with Neurons

**DOI:** 10.3389/fnagi.2016.00116

**Published:** 2016-06-27

**Authors:** Tamara Logica, Stephanie Riviere, Mariana I. Holubiec, Rocío Castilla, George E. Barreto, Francisco Capani

**Affiliations:** ^1^Laboratorio de Citoarquitectura y Plasticidad Neuronal, Facultad de Medicina, Instituto de Investigaciones Cardiológicas Prof. Dr. Alberto C. Taquini (ININCA), UBA-CONICET, CABABuenos Aires, Argentina; ^2^Laboratorio de Biología Molecular, Facultad de Medicina, Instituto de Investigaciones cardiológicas Prof. Dr. Alberto C. Taquini (ININCA), UBA-CONICET, CABABuenos Aires, Argentina; ^3^Departamento de Nutrición y Bioquímica, Facultad de Ciencias, Pontificia Universidad Javeriana BogotáBogotá, Colombia; ^4^Departamento de Biología, Universidad Argentina JF KennedyBuenos Aires, Argentina; ^5^Investigador Asociado, Universidad Autónoma de ChileSantiago, Chile

**Keywords:** perinatal asphyxia, brain, metabolism, neuron, astrocyte, interaction

## Abstract

Perinatal Asphyxia (PA) represents an important cause of severe neurological deficits including delayed mental and motor development, epilepsy, major cognitive deficits and blindness. The interaction between neurons, astrocytes and endothelial cells plays a central role coupling energy supply with changes in neuronal activity. Traditionally, experimental research focused on neurons, whereas astrocytes have been more related to the damage mechanisms of PA. Astrocytes carry out a number of functions that are critical to normal nervous system function, including uptake of neurotransmitters, regulation of pH and ion concentrations, and metabolic support for neurons. In this work, we aim to review metabolic neuron-astrocyte interactions with the purpose of encourage further research in this area in the context of PA, which is highly complex and its mechanisms and pathways have not been fully elucidated to this day.

## Introduction

Hypoxia-Ischemia (HI) in the human newborn, also called hypoxic-ischemic encephalopathy or perinatal asphyxia (PA) is the interruption of gas exchange and blood flow to the fetus in the perinatal period (Volpe, 2001) and remains a serious complication with a high mortality and morbidity rate (van Bel and Groenendaal, 2008). It is usually caused by intrauterine asphyxia—antepartum or intrapartum, and it derives in a great range of non-progressive neurological deficits (Volpe, 1976). During the first days, the encephalopathy caused by neonatal brain injury, is manifested by signs evolving from lethargy to hyper-excitability to stupor (Sarnat and Sarnat, 1976). As a consequence of PA approximately 45% of newborn die and 25% have permanent neurological deficits, including developmental delay, various motor deficits—often clustered as “cerebral palsy”—with or without mental retardation (Vannucci and Perlman, 1997), learning deficits or disabilities (Gunn, 2000), visual and hearing problems (Osborne et al., 2004), and various issues regarding school readiness (Shankaran, 2009; Romero et al., 2014). Despite the great advances made in obstetric and neonatal care during the last decades no effective treatment for PA brain injury has been developed.

Although an adult brain is composed by approximately 100,000 million neurons, astrocytes—a non-excitable type of cell—outnumber them by over fivefold, thus representing the most numerous cell type within the whole central nervous system (CNS) (Kandel, 1970). Over the past century, since Ramón y Cajal’s histological studies took place and until nowadays, neurons have been considered the morphological and functional unit of the CNS (García-Marín et al., 2007; Navarrete and Araque, 2014). For over a century astrocytes were given a passive role in the CNS in which their function was regarded mainly as support for neurons. It was only after electron microscopy advances, when studies were focused in the morphology of synapses, that their crucial role in the CNS was discovered. Critical functions essential for the neuron to normally function, were finally acknowledged (Markiewicz and Lukomska, 2006; Kimelberg and Nedergaard, 2010).

Not many decades ago it was discovered that astrocytes not only represent the main source of nutrients and growth factors for neurons, but that they have a strategic localization that enables intimate communication between them and neurons (Halassa et al., 2007; Fiacco et al., 2009). This relationship is based not only in physical proximity, but also in a highly complex and fast intra and intercellular signaling system. Both cell types are in continuous collaboration and these signaling pathways make possible the intimate communication astrocytes share with neurons that allows them to perform many critical functions (Kirchhoff et al., 2001). An exhaustive metabolic exchange occurs between these type of cells, for example astrocytes express enzymes involved in several metabolic pathways that neurons cannot express (Kirchhoff et al., 2001; Volpe, 2001). Gap junction channels allow both types of brain cells to form a large syncytium where astrocytes release several neuroactive substances (Piet et al., 2004). Astrocytes also have a large amount of neuro-transmitter receptors that allow them to maintain a fluid communication with neurons (Sattler and Rothstein, 2006; Seifert et al., 2006).

The normal function of this interaction in healthy conditions promotes a constant and dynamic communication between neurons and astrocytes, enabling them to influence and affect each other in many ways. In this sense, understanding the damage mechanisms of hypoxia, taking both cellular types separately, results inconceivable. Along these lines, during disease, this fluid communication can translate either in cooperation in order to restore homeostasis, or the propagation of damage (Hatten et al., 1991; Chen and Swanson, 2003; Ferriero, 2004; Floyd and Lyeth, 2007; Jeong et al., 2014; Romero et al., 2014). It has been shown that most of the injury in the neonatal brain caused by PA is mainly due to a metabolic imbalance (Ferriero, 2004). The role of astrocytes and their symbiotic interaction with neurons is to create a metabolic unit essential in the course of the injury. Taking into account this relationship, but specially the role of astrocytes seems to be crucial, not only to understand the several mechanisms underlying the damage caused by PA, but also to develop therapeutic tools to counteract the aforementioned devastating consequences. Indeed, astrocytes appear to be more resistant to damage than the rest of the cells present in the CNS and several studies even suggest that they play a beneficial and essential role when brain insult occurs (Desagher et al., 1996; Blanc et al., 1998; Ye and Sontheimer, 1999; Ahlemeyer et al., 2000).

In this article, we will summarize the mechanisms underlying the damage caused by PA, as well as recent advances in the knowledge gained about the crucial role of astrocytes in their relationship with neurons. We will also revise metabolic neuron-astrocyte interactions and their role in PA brain injury in order to promote further understanding of these processes as well as considering this neuron-astrocyte relationship when developing neuroprotective tools to counteract the devastating consequences that PA causes in children.

## Perinatal Asphyxia: An Injury in the Immature Brain

The latest advances in the study of brain injury in neonates during the past decades seem to confirm how erroneous it is to conceive brain injury as a uniform event. On the contrary, several recent studies have shown that neonatal brain injury is not a consequence of a the primary insult due to PA, it has been revealed that the cause is protean, and mainly metabolic, in terms of energy failure during the re-oxygenation and transient ischemia-reperfusion, but also that the brain injury caused by PA can be due to defects in inherited metabolic pathways (Ferriero, 2004). Several studies have shown that the mechanisms underlying the pathogenesis of the HI injury are rather complex in the immature brain and they are determined by many variables, such as maturity level of the brain, timing and intensity of asphyxia, selective vulnerability of the various regions in the brain, the severity of the insult, and as aforesaid, the evolution of the re-oxygenation phase (Walton et al., 1999; Ferriero, 2004; Sugawara et al., 2004; Alvarez-Díaz et al., 2007; Romero et al., 2015).

Throughout the mammalian clade, the brain requires a permanent supply of oxygen and nutrients, especially during development, when the brain needs large amounts of energy (Chugani and Phelps, 1986; Takahashi et al., 1999). In the adult brain, glucose is almost mandatory as an energy substrate, whereas oxidative metabolism of glucose is also the most efficient pathway during development; the immature brain has the capacity to utilize other substrates, including ketone bodies, lactate, fatty acids and amino acids for energy and biosynthesis of lipids and proteins (Nehlig, 1997, 2004; Chowdhury et al., 2007; Brekke et al., 2015). The substrates required to obtain energy are transported via blood flow (Settergren et al., 1976). But when PA occurs, energy levels fall immediately due to deprivation of glucose and oxygen, and this constitutes an hypoxic-ischemic event that affects brain maturation with long-lasting consequences for the adult brain (Vannucci and Hagberg, 2004).

When a hypoxic-ischemic insult occurs, a mechanism of neuronal injury known as excitotoxicity is unchained. Neuronal glutamate (Glu) metabolism, which consists in the cycle of Glu release, reuptake and re-synthesis, is the main metabolic pathway in the brain and it is coupled to cerebral glucose oxidation (Sibson et al., 1998a,b). Removal of Glu from the extracellular space depends on glial cells. Astrocytes cannot re-uptake the Glu due to the lack of ATP, leading to high synaptic Glu levels (Magistretti et al., 1999). During ischemia the intracellular calcium levels present a significant rise, these high abnormal concentrations activate calcium dependent proteases, lipases and DNAses. Due to the lack of energy, the cell cannot perform the synthesis of the previously damaged constituents (Doyle et al., 2008).

A primary cell death is produced by glucose and oxygen deprivation, paving the way for a more devastating secondary cell loss due to reperfusion and re-oxygenation (Inder and Volpe, 2000). In normally functioning mitochondria, more than 80% of oxygen in the cell is reduced to energy equivalents (ATP) by cytochrome oxidase but the rest is converted to superoxide anions. These anions will be reduced to water by enzymatic and non-enzymatic antioxidant mechanisms in normal conditions. In a hypoxic-ischemic event a damage to the energy-producing machinery of the mitochondria is created, the result is an accumulation of superoxide and a conversion of superoxide to other reactive species such as the hydroxyl radical (Capani et al., 2001, 2003; Ferriero, 2001). When re-oxygenation occurs, the cells have been damaged by hypoxia, mitochondrial oxidative phosphorylation is overwhelmed and reactive oxygen species (ROS) accumulate (Ferriero, 2001). The membrane and pump function are altered as a consequence of free radicals’ accumulation, allowing more Glu release leading to more excitotoxicity. In addition, the accumulation of free radicals produces activation of pro-apoptotic mediators, and direct DNA and protein damage. Several antioxidant enzymes, superoxide dismutase, catalase, and glutathione peroxidase are part of the antioxidant defense system. In the immature brain of rats, these enzymes, display less activity compared to the adult brain, this may reinforce the vulnerability of the neonatal brain to redox imbalance (Khan and Black, 2003).

## Astrocytes, General Considerations

As stated above, astrocytes, the main glial cell type found in the CNS, generally outnumber neurons by over fivefold (Sofroniew and Vinters, 2010). During the last five decades astrocytes have been classified into two major categories, depending on their morphology and spatial organization: fibrous and protoplasmic (Haycock and Bro, 1975). Fibrous astrocytes, are found in white matter and compared with protoplasmic astrocytes, they have fewer but longer and larger unbranched processes that contact nodes of Ranvier (Haycock and Bro, 1975). Protoplasmatic astrocytes, present in gray matter, have shorter and numerous processes in a uniform globoid distribution, enveloping synapses (Haycock and Bro, 1975). Both types of astrocytes form gap junctions between distal processes of adjacent astrocytes, this is a specialized contact that allows the direct transfer of small molecular weight molecules between them (Peters, 2007). The traditional categorization also includes another type of astrocytes known as radial astrocytes. Unlike neurogenesis, gliogenesis remains active after birth in rats and radial glia that are precursors of the astroglial linage and are oriented at right angles to the ventricular surfaces and have elongated and unbranched processes (Malatesta et al., 2008). After neonatal HI, these cells present an abnormal morphology and architecture, and it has been found that they rapidly transform into astrocytes right after the insult and over several weeks in the cortex of rats (Sizonenko et al., 2008).

Certain proteins, such as glial fibrillary acidic protein (GFAP), Glutamine Synthetase (GS) and S100β, expressed by astrocytes are used as markers for immunohistochemical identification of this cell type (Norenberg, 1979; Goldman, 2003; Gonçalves et al., 2008). GFAP, a member of an intermediate filament protein family, is involved in maintenance of white matter in the CNS as well as in the integrity of the blood brain barrier (BBB) (Liedtke et al., 1996; Kakinuma et al., 1998). Although both types of astrocytes express GFAP, protoplasmic astroglia does not express enough protein to stain positive with routine immunohistochemical methods (Walz, 2000a,b). In previous studies using transgenic mice, this protein has been proven to be essential for the process of reactive astrogliosis and glial scar formation while it seems to play no part in normal morphology and function of most astrocytes in healthy CNS (Pekny et al., 1995; Herrmann et al., 2008). During astrogliosis, which occurs subsequently to trauma, astrocytes proliferate, swell, and undertake fibrosis by the accumulation of filaments, expressed as an increase in GFAP and/or the novo expression of vimentin, the latter being the main feature of this process (Bauer et al., 2007). Furthermore, S100β, leaks from astrocytes into the blood after acute hypoxemia in the case of BBB disruption (Giussani et al., 2005). Thereby, increased plasma levels of this protein are considered as a good marker for different brain injuries (Persson et al., 1987; Blennow et al., 2001). After leakage, cells do not increase its expression, suggesting a reduction of S-100 in brain cells (Goni-de-Cerio et al., 2009).

## Anatomical Organization

In the CNS, there are no regions bare of astrocytes. The distribution of this type of cells is orderly and well organized in a contiguous and essentially non-overlapping manner. Some anatomical studies have demonstrated that individual protoplasmatic astrocytes in gray matter have non-overlapping domains in the healthy CNS (Bushong et al., 2002; Ogata and Kosaka, 2002; Halassa et al., 2007). Thus, only the most distal tips of processes from individual astrocytes interlace with one another where the gap junctions are formed. The diffusion of molecules among them increase the communication between adjacent astrocytes (Bushong et al., 2002, 2004; Ogata and Kosaka, 2002; Nedergaard et al., 2003; Halassa et al., 2007). Studies in cortical and hippocampus astrocytes showed that these cells do not have overlapping territories and that a single astrocyte process enwraps from four to eight neuronal *somata*, hundreds of dendrites and enfold more than 100,000 synapses (Bushong et al., 2002; Ogata and Kosaka, 2002; Halassa et al., 2007). Therefore, small bunches of neurons could be coordinated by one astrocyte that contacts them to their *somata*. In contrast, when an astrocyte transmits signals to dendrites or synapses it regulates hundreds of neurons which *somata* are away from each other, this is why astrocytes play a decisive role in neural circuit tasks, in synaptic transmission and information processing (Bushong et al., 2002; Halassa et al., 2007). This tight relationship between neurons and astrocytes is bidirectional; in fact it has been shown that alterations in gap junctions due to connexins 43 and 30 cause demyelinization in neurons (Lutz et al., 2009).

## Astrocytes’ Main Functions and their Intimate Interaction with Neurons in the Immature Brain

### Development: Cell Migration, Synapses Development and Pruning

During development, astrocytes act as a guide and support for post-mitotic neuron migration. Radial glia express certain receptors, as metabotropic Glu receptor 5, that play a key role in promoting the extension of these cell processes, thus regulating the migration of cortical neurons during development. Moreover, the activation of this receptor promotes the survival and proliferation of progenitor cells (Jansson and Åkerman, 2014). Although astrocytes have a close relationship with neurons during development, guiding and controlling neuronal networks, they also play a key role in biochemical regulation of the development and growth of axons and dendrites. They form molecular boundaries that play an important role in axon growth and dendrites (Powell and Geller, 1999). Growing axons are guided towards their target by molecules, like some proteoglycans and the glycoprotein tenascin C, which are, in fact, derived from astrocytes (Powell and Geller, 1999).

In the adult brain, astrocytes have the same function when it comes to axonal regeneration and synaptogenesis. Indeed, a study with ganglionic cells from the retina, demonstrated that in the absence of glia, neurons showed poor synaptic activity, but in presence of astrocytes, and not other cell types, it increased at least 100 times (Pfrieger and Barres, 1997; Ullian et al., 2001). It is worth to mention that another study showed a rise in the amount of synapsis by 7 times when ganglionic cells were co-cultured with astrocytes; this may explain the astrocyte-mediated increase of synaptic activity (Ullian et al., 2001). This increase is mediated by the correct release of thrombospondins, a family of proteins expressed by astrocytes during development, but also as a consequence of brain damage later in life, which are responsible for synaptogenesis and are involved in correct synapse functioning and development (Christopherson et al., 2005).

Astrocytes also release specific signals involved in synaptic pruning related to the development and function of synapses (Barres, 2008). For example, astrocytes are responsible for inducing C1q expression in synapses to tag them for microglial removal at developmental stages (Stevens et al., 2007). The involvement of astrocytes in synaptic pruning is also mediated by the phagocytic pathway Draper/Megf10 and Merk/integrinalpha(v)beta5 (Barres, 2008).

### Tripartite Synapses

One of the most notorious advances regarding the knowledge about astrocyte functions led to the concept of the tripartite synapsis, developed as a consequence of the finding that, not only their excitability, based in variations in intracellular Ca^2+^ concentrations, can be regulated by neurons with certain neurotransmitters release (Cornell-Bell and Finkbeiner, 1991), but also that neurons’ synapsis transmission can be regulated, as well, by Glu released from astrocytes in a Ca^2+^ dependent manner (Araque et al., 1999; Rusakov et al., 2011; Nedergaard and Verkhratsky, 2012). From a morphological point of view, astrocytes and neurons are strategically placed as a unit in the synapses, in order to maintain a close interaction, since both pre- and post-synaptic zones are in close contact with astrocytic foot processes, which suggests that astrocytes might be contributing to information processing in neurons (Araque et al., 1999; Halassa et al., 2007).

An increase in astrocytic cytosolic Ca^2+^ occurs as a consequence of neuronal activity. There are two different types of Ca^2+^ signaling modalities: oscillations and propagating waves of this divalent cation (Cornell-Bell and Finkbeiner, 1991). Ca^2+^ oscillations are augments of the intracellular concentrations of this ion limited to a single cell, provoked by exposure to different transmitters, such as ATP, Glu and GABA (Charles et al., 1991). They can also be triggered by extracellular Ca^2+^ removal. Propagating Ca^2+^ waves can be boosted by application of focal electrical or mechanical stimulation (Charles et al., 1991).

The other, Ca^2+^ signaling modality is propagating Ca^2+^ waves. This can be stimulated by local application of transmitters such ATP or Glu, lowering extracellular Ca^2+^ levels, focal electrical or mechanical stimulation. Astrocytic Ca^2+^ waves are induced by elevated neuronal spiking frequency. This was shown in anaesthetized rodents exposed to subsequent sensory stimulation (Sanderson et al., 1990; Charles et al., 1992). When astrocytes achieve a certain level of activation, ATP will be released and it will provoke Ca^2+^ increases in neighboring astrocytes, resulting in a spatial expansion of astrocytic activation. This is why Ca^2+^ waves could be seen as a means of astrocytic activation amplification.

To bear in mind the concept of tripartite synapses when studying this interaction during PA becomes crucial, since it was demonstrated that this relationship is characteristic of the developmental period. Indeed, in astrocytes, the mGluR5, which mediates the triggering of Ca^2+^ waves, is developmentally regulated and peaks at postnatal day 7 in rodents (Araque et al., 1999; Panatier et al., 2011). In fact, this receptor is the main metabotropic Glu receptor and is expressed in progenitors and radial glia cells (Di Giorgi Gerevini et al., 2004). Sun et al. (2013) found no expression of mGluR5, in murine astrocytes after postnatal day 3, and neither in human astrocytes from cortical and hippocampal tissue in adults, suggesting that astrocytic Ca^2+^ signaling evoked by synaptic release of Glu may thus be confined to the young rodent pups. Also, the hippocampus, one of the most vulnerable areas of the brain that suffers the consequences of PA has at least 57% of the synapses has had astrocytic processes opposed to them (Ventura and Harris, 1999).

As previously discussed, the conception of astrocytes as static and isolated units has been cast down given a recent and great body of evidence demonstrating how the highly dynamic and complex interaction between neurons and astrocytes contribute, not only to a healthy functioning of the CNS under physiological conditions, but also, to survival and resistance in pathophysiological circumstances. Their intimate interactions range from the very development of the CNS, in which their relationship makes possible cell migration, synapses development and pruning, to the complex metabolic interaction enabled by the strategic placement of both cell types.

### Neurotransmission

In order to clear neurotransmitters from the synaptic space, astrocytes express in their processes high levels of transporters for neurotransmitters, such as Glu, GABA, and glycine (Sattler and Rothstein, 2006; Seifert et al., 2006). Glu uptake is probably the most important in this regard. After the release of Glu from glutamatergic neurons, this compound must be inactivated in order to avoid excitotoxicity. Whereas neurons also recycle Glu via reuptake of this neurotransmitter, this process is a pathway necessary for its reutilization (Hertz et al., 1999; Daikhin and Yudkoff, 2000). This is accomplished through cellular uptake of the neurotransmitter from the synapsis (Clements et al., 1992), a task performed mostly by astrocytes (Pellerin and Magistretti, 1994; Bergles and Jahr, 1998). A synchronized *de novo* synthesis of amino acid transmitters takes place in neurons, and astrocytes make the intermediator available for this cycle to be accomplished. Their assistance to neurons in this task results imperative, since Glutamine Synthetase (GS), the enzyme responsible for Glu amidation into glutamine, is present only in astrocytes and not in neurons (Norenberg and Martinez-Hernandez, 1979). Glutamine has no neurotransmission purpose so it can be safely released from astrocytes for rapid neuronal uptake in order to complete Glu regeneration in the neuron (Kvamme et al., 2000); (For more information refer to “Glutamate Uptake by Astrocytes” Section).

This process leads to several changes in astrocyte metabolism, due to the fact that Glu enters into astrocytes with co-transportation of Na^+^, while K^+^ is extruded (Brew and Attwell, 1987), affecting astrocytic metabolism since intra-astrocytic Na^+^ concentrations increases and the Na^+^/K^+^ ATPase is activated. The above mentioned processes result in further stimulation of glycolysis and thus, glucose consumption that subsequently generates lactate production (Chatton et al., 2000; Magistretti and Pellerin, 2000).

### Gliotransmition

It has been previously shown that astrocytes can exhibit excitability due to neuronal or self-generated stimulation, and thus are able to modulate synaptic transmission and excitability (Araque et al., 2001; Volterra and Meldolesi, 2005; Harada et al., 2015). Araque et al. (1998) demonstrated that when an astrocyte is activated its intracellular Ca^2+^ levels rise, as a result of Ca^2+^ release mainly from the endoplasmic reticulum, causing the release of Glu and a delayed Ca^2+^ rise in nearby neurons. During the following decade different components of this alleged exocytotic machinery in astrocytes were discovered and identified, giving more strength to the hypothesis of gliotransmission. Processes related to Ca^2+^ dependent release, a synaptic-like microvesicle (SLMV) which is crucial for Glu release and exocytotic fusion were identified over the years (Volterra and Meldolesi, 2005; Harada et al., 2015).

Gliotransmission mechanisms have been proven to be quite complex. For instance, it has been shown that different stimuli that generate a Ca^2+^ dependent response, cause diverse gliotransmitter release responses (Volterra and Meldolesi, 2005). Moreover, non exocytotic gliotransmitters release has been described. Indeed, gliotransmitters, particularly ATP and Glu, can be released from volume regulated ion channels, gap-junction hemmichanels and purinergic P2X receptors (Volterra and Meldolesi, 2005).

### Blood Brain Barrier

As previously mentioned, astrocytes play a key role in the supply of certain metabolites that are essential for the developing brain to function adequately, but this process starts with the transport of these nutrients to the brain. An appropriate supply of nutrients from circulation is assured by the BBB, which is formed by various types of cells: pericytes, endothelial cells, basal lamina and astrocytes (Engelhardt, 2003). All these cells are arranged as a complex unit in charge of maintaining homeostasis of low permeability thus supplying a wide range of nutrients such as glucose, amino acids, monocarboxylates and vitamins, essential for the proper development and functioning of the brain (Møllgård and Saunders, 1986; Saunders et al., 1991, 2016). This permeability depends mainly on a complexly specialized endothelial cell layer of the brain whose phenotype differs from the cells that form capillaries beyond the brain (Pries et al., 2000). The essential role of astrocytes regarding metabolic transport between the BBB and neurons lays in their close contact with brain capillaries and endothelial cells, since this is what allows them to be in direct contact with energy substrates and metabolites that are transported from the blood to brain cells.

During development, radial glia participates in the expression of proteins that form the tight junctions of microvessels. Astrocytes end-feet inductive influence is also essential for the complex tight junctions that make possible the specialization of BBB endothelium phenotype (Janzer and Raff, 1987).

Astrocytes also play a key role in water homeostasis, their processes count with a large amount of aquaporin water channels (Zador et al., 2009) densely clustered, making contact with blood vessels, and maintaining fluid homeostasis. Hence, when an ischemic injury occurs, they play a strategic role regarding water imbalance, brain swelling and thus, the progress of edema itself (Kimelberg, 2005).

### Blood Vessels and Blood Flow

**Figure 1:**
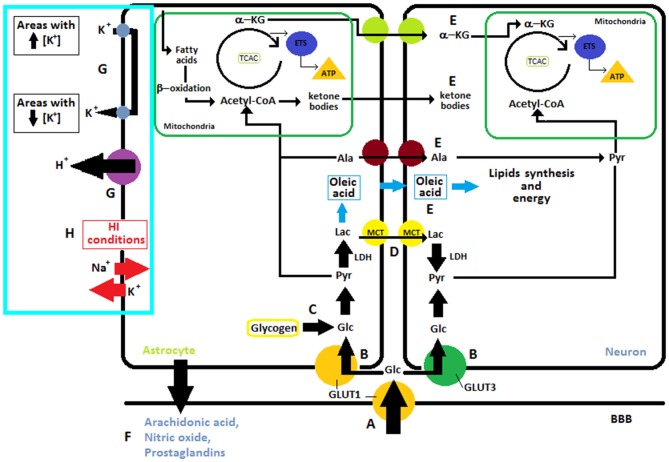
**I. Overview of different substrates obtained from glucose: (A)** Glucose is transported from capillaries through GLUT1 and **(B)** into the neuron via GLUT3 or into the astrocytes through GLUT1. **(C)** In astrocytes glucose can also be obtained from glycogen reserves to then be transformed into pyruvate which enters into the mitochondria where cellular respiration takes place. The neuron can utilize glucose from blood or lactate, **(D)** transported from the astrocytes to get the pyruvate. **(E)** Neurons can also uptake other metabolites like alanine, oleic acid, ketone bodies and α-ketoglutarate from astrocytes. **II**. Regulation of the blood flow through the release of molecular mediators by astrocytes **(F)**. **III**. Ions and K^+^ Uptake. **(G)** In normal conditions, astrocytes are involved in “spatial buffering” of K^+^ and proton shuttling, for this purpose they have Na^+^/H^+^ exchangers, bicarbonate transporters, monocarboxylic acid transporters, and the vacuolar-type proton ATPase in their membrane. **(H)** After ischemia, lack of energy leads to the release of potassium into the extracellular space and entry of sodium into cells, this causes neuronal plasma membrane depolarization. Abbreviations: ETS, Electron Transport System; a-KG, α-ketoglutarate; LAC, Lactate; Pyr, Pyruvate; TCAC, Tricarboxylic acid cycle; LDH, Lactate Dehydrogenase; ALA, Alanine; GLC, Glucose; K^+^, potassium; Na^+^, sodium; H^+^, proton.

One of the hallmarks of brain energy metabolism is the neurovascular coupling. A great body of evidence supports the relationship between a local neuronal activation and the ensuing changes in local blood flow in the brain, implicating a wide variety of vasoactive agents such as H^+^, K^+^, several neurotransmitters, adenosine, arachidonic acid, metabolites and nitric oxide (Gordon et al., 2008; Carmignoto and Gómez-Gonzalo, 2010; Gómez-Gonzalo et al., 2010). Astrocytes are strategically positioned to both, sense neuronal activity in the synapse and, with their end-feet enwrapped in blood vessels, adequately respond with the corresponding metabolic supply. Several studies evidence the key role of astrocytes in the changes of blood flow related to neuronal activity, not only vasoconstriction but also vasodilation, and especially through their interaction with neurons and Glu signaling (Takano et al., 2006; Iadecola and Nedergaard, 2007; Koehler et al., 2009; Attwell et al., 2010; Carmignoto and Gómez-Gonzalo, 2010). Some of these studies also show the part astrocytes take in these processes through the release of molecular mediators—such as arachidonic acid, nitric oxide and prostaglandins- that help regulate the blood flow in the CNS by increasing or decreasing blood vessels diameter (Figure 1) (Gordon et al., 2007; Iadecola and Nedergaard, 2007). It has also been shown via fMRI that the proper function of astrocytes is essential to respond to visual stimuli in the visual cortex (Schummers et al., 2008; Wolf and Kirchhoff, 2008). Astrocytes had been shown to play a great part in neurovascular coupling as important intermediates in neuronal signaling to blood vessels. Hence, regarding neurovascular coupling, our understanding of astrocytes functions is challenging neurocentric paradigms to integrate the dynamic cooperation with neurons.

### Ions and K^+^ Uptake

It is well known that astrocytes Figure 1 play a key role in maintaining homeostasis in the extracellular environment, spatially buffering ions such as K^+^ (Leis et al., 2005). Astrocytes processes are rich in transporters utilized for K^+^ uptake (Simard and Nedergaard, 2004; Seifert et al., 2006). Under normal circumstances, astrocytes are able to take up K^+^ from extracellular areas with high concentration of this ions and redistribute it into their own cytoplasmic volume and gap junction syncytium, in order to posteriorly release it in regions with lower K^+^ concentrations. In this fashion, astrocytes are in charge of maintaining homeostasis in extracellular space through a “spatial buffering” of K^+^ (Leis et al., 2005). Moreover, astrocytes are intimately involved in proton shuttling, for this purpose they have Na^+^/H^+^ exchangers, bicarbonate transporters, monocarboxylic acid transporters, and the vacuolar-type proton ATPase in their membrane (Obara et al., 2008). After ischemia, ATP is consumed within 2 min, these lack of energy leads to the release of potassium into the extracellular space and entry of sodium into cells which causing neuronal plasma membrane depolarization (Figure 1) (Caplan et al., 1990).

## Metabolic Interaction Between Astrocytes and Neurons

In the feat of delivering sufficient energy to sustain the high requirements of the brain, it becomes hard to conceive glucose as the mandatory and single metabolic substrate. Indeed, ketone bodies are essential during development and starvation (Nehlig, 2004; Magistretti, 2008). Glycogen, reserved in astrocytes, can be used throughout periods of elevated neuronal activity and hypoglycemia, and lactate when performing intense physical activity (Jeppesen et al., 2009). Furthermore, different types of cells, under different circumstances, can efficiently exploit other substrates or glucose intermediates, such as lactate, pyruvate, Glu or acetate (Zielke et al., 2009). Some of the aforementioned substrates, such as glucose or some monocarboxylates, are transported from circulation into the brain by proteins belonging to two integral membrane protein families: (1) GLUTs, glucose transporters and (2) MCTs, proton-coupled monocarboxylic acid transporters (Dwyer et al., 2002; Vannucci and Vannucci, 1997) (Figure 1).

Nowadays the fact that astrocytes Figure 1 make crucial contributions to the CNS metabolism has become common knowledge. This type of cell is well positioned to take up glucose from blood vessels and provide energy metabolites to different neural elements in gray and white matter. Their processes contact blood vessels, synapses, neuronal *perikarya* and axons, at nodes of Ranvier (Sofroniew and Vinters, 2010). Furthermore, astrocytes are main storage sites of glycogen granules in the CNS (Figure 1) (Phelps, 1972). A profound understanding of the many metabolic pathways in the brain challenges the limitations of the neuron-centered point of view of neuroenergetics and metabolism leading to the concept of metabolic cooperation between these two types of cells.

### Glucose

There are at least two different ways of obtaining glucose: (1) by transportation from blood vessels entering into the astrocytes through GLUT1 or into neurons through GLUT3 and (2) by hydrolysis of glycogen, which is localized predominantly in astrocytes (Chen and Swanson, 2003). In both cases, pyruvate is acquired via glycolysis. The lactate dehydrogenase converts pyruvate into lactate which is shuttled to neurons via MCT1 (Chen and Swanson, 2003). As shown in Figure 1 neurons can uptake other metabolites also like alanine and α-ketoglutarate from astrocytes (Chen and Swanson, 2003).

Astrocytes play an important role supplying energy substrates to neurons, a wide range of metabolic intermediates formed from glucose in the brain can subsequently be oxidized for energy production and depend basically on oxygen (e.g., lactate, pyruvate, Glu, or acetate). For that reason, during neonatal HI the neuronal function cannot be sustained despite of the increase of GLUTs overexpression observed in immature rat brain in this pathological condition. (Vannucci et al., 1998).

The expression of GLUT1 in the BBB and the enzymes of the tricarboxylic acid cycle are higher in the adult brain than in the developing brain because glucose is the almost exclusive fuel in the adult brain but not in the immature brain (Vannucci and Simpson, 2003). The expression of this transporter rises abruptly in the second postnatal week in rats (Vannucci et al., 1993).

### Role of Lactate in Developing Brain

**Figure 2:**
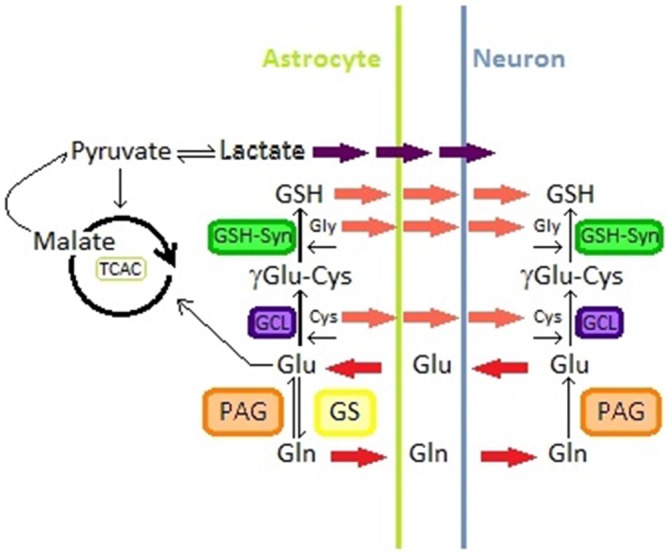
**Glutamine-glutamate pathway in astrocytes and neurons; Glutathione synthesis in astrocytes and neurons; lactate production from malate in astrocytes.** Abbreviations: Glu, Glutamate; Gln, Glutamine; GS, Glutamine Synthetase; PAG, phosphate-activated glutaminase; TCAC, tricarboxylic acid cycle; GSH, gluthation; Cys, Cysteine; Gly, Glycine; GCL, glutamine Cys ligase; GSH-syn, GSH synthetase.

Lactate is a significant metabolic substrate for the brain. Throughout adult life, and particularly during a starvation situation, lactate is used as an energy source by brain cells. Astrocytic glycogen is broken down and, in this way, lactate is obtained (Figure 2). Lactate is an exchangeable substrate among astrocytes and neurons that works maintaining energy homeostasis in the CNS (Medina and Tabernero, 2005). During the perinatal period, lactate plays a very important part, it is the main substrate during brain development and all brain cell types use it as a precursor of lipids and energy source. This premise regarding lactate as the main source of energy for neurons is the foundation for the “lactate shuttle hypothesis” that postulates the idea that glial cells, especially astrocytes, transform glucose into lactate which is then transported into the neurons to be their metabolic source (Pellerin et al., 2007). Astrocytes use lactate and other metabolic substrates to synthesize oleic acid—which is the only fatty acid synthesized by these cells and acts as a neurotrophic factor (Figure 1) (Tabernero et al., 2001; Rodríguez-Rodríguez et al., 2004). Neurons and oligodendrocytes use it as an energy source and as a precursor of lipids. Regarding oligodendrocytes, they use oleic acid for myelin synthesis, while neurons use lactate as energy source for differentiation and proliferation. In other words, lactate is an essential substrate in the developing brain, such as glucose is in the adult brain. It is worth to mention that in the immature brain, compared to the adult one, lactate transport across the BBB is higher because of the predominant expression of MCTs (Cremer, 1982; Conn et al., 1983; Pellerin et al., 1998b; Fayol et al., 2004). In the intrauterine period lactate is transported to the fetus throughout placental membranes. The transplacental supply of nutrients finishes with a phase of postnatal starvation, which is the pre-suckling period that is followed by a fat-rich diet adaptation. After delivery an increase in fatty acid concentrations occurs, this is due to triacylglycerol breakdown. In human newborns fatty acids are obtained from white adipose tissue. In rats, because of the lack of white adipose tissue at birth, they come from hydrolysis of triacylglycerols from the mother’s milk. Nonetheless, in both species, once lactation is active, fatty acids come from the intestinal hydrolysis of milk triacylglycerols (Aw and Grigor, 1980). Despite the fact that there is a rapid exchange of oxygen between maternal and fetal blood, the rate of oxygen usage in the fetus is moderate when compared to the one measured in the newborn during the neonatal period (Battaglia and Meschia, 1978; Girard et al., 1981). This boost in oxygen accessibility may well trigger the use of lactate (Medina, 1985), thus starting postnatal energy homeostasis. Later on, in the suckling period, ketone bodies become the main fuel for the brain and lactate is used as the major gluconeogenic substrate (Fernández and Medina, 1986).

### Astrocyte—Neuron Lactate Exchange

In astrocyte cultures it was observed that this type of cells produce lactate from extracellular glucose (Walz and Mukerji, 1988). These results suggest that astrocytes may convert blood glucose into lactate through glycolysis. In addition, synaptic activity seems to regulate this process, astrocytic glycolysis is modulated with lactate necessities of neurons (Bouzier-Sore et al., 2002). Lactate can pass through the plasma membrane and be accessible to neurons (Pellerin et al., 1998b). Then, lactate is transported to the neuron cytoplasm by specific carriers (Figure 2) (Dringen et al., 1993; Tildon et al., 1993; Debernardi et al., 2003).

Throughout the postnatal period, glucose is scarce but, as already stated, lactate is used by both neurons and astrocytes to maintain brain development. Glucose remainders are used by the brain cells for production of nicotinamide adenine dinucleotide phosphate (NADPH) and glycerol-borne phospholipids (Tabernero et al., 1996).

The astrocytic and neuronal ability to use lactate in order to save glucose for those purposes that lactate itself cannot fulfill, might be the reason why this molecule is used in hypoglycemia periods (Schurr et al., 1988; Bock et al., 1993; Izumi et al., 1994).

### Ketone Bodies

Originally, the liver was thought to be the major, if not the only organ, capable of supplying the brain with ketone bodies (e.g., 3-hydroxybutyrate and acetoacetate) from fatty acids, but not many decades ago it was discovered the ketogenesis capacity of astrocytes not only from fatty acids, but also from leucine (Auestad et al., 1991; Bixel and Hamprecht, 1995). Furthermore, both, astrocytes ketogenic system resemble ketone bodies production by hepatocytes, indicating both cell types share similar properties (Guzmán and Blázquez, 2001).

As well as lactate supply from astrocytes is enhanced in situations of high oxidative metabolism requirements for the neurons, such as synaptic activity (Wiesinger et al., 1997; Pellerin et al., 1998a; Deitmer, 2000; Tsacopoulos, 2002), it has been found that Glu can enhance ketogenesis in astrocytes via Glu transporters (Guzmán and Blázquez, 2004).

Under hypoxic conditions, anaerobic metabolism is stimulated in astrocytes and lactate production is enhanced and this molecule accumulates (Dienel and Hertz, 2001). An AMP- activated protein kinase (AMPK) is activated in astrocytes in response to metabolic stress, which phosphorylates and inactivates acetyl-CoA carboxylase, thereby decreasing malonyl-CoA concentration (Blazquez et al., 1999). Therefore, carnitine palmitoyltransferase I (CPT-I), a mitochondrial outer membrane enzyme, is released from inhibition and can increase the supply of fatty acid substrates and the later production of ketone bodies from β-oxidation in the mitochondrial matrix which are shuttled from astrocytes to neurons (Figure 1) (Guzmán and Blázquez, 2004). In neuron cultures, the 3-hydroxybutyrate was preferred over lactate as energy substrate in hypoxic conditions (Blazquez et al., 1999). There is a debate about if ketone bodies may replace glucose as an energy substrate to preserve neuronal function and its neuroprotective role in hypoglycemia or HI (Arakawa et al., 1991; Izumi et al., 1998; Brown et al., 2001; Massieu et al., 2001; Guzmán and Blázquez, 2004).

### Glycogen

As previously stated, astrocytes are the main storage sites of glycogen granules in the CNS and maximum accumulation of this substrate occurs in areas of high synaptic density (Phelps, 1972). The content of glycogen can be modulated by neurotransmitters (Brown and Ransom, 2007). The utilization of this glycogen can maintain neuronal activity throughout periods of elevated neuronal activity and hypoglycemia. Under conditions of hypoglycemia, there is evidence that indicates that astrocytic glycogen breaks down into lactate and is transferred to neighboring neural elements where it is used aerobically as a carbon source (Voutsinos-Porche et al., 2003; Brown et al., 2004; Brown and Ransom, 2007; Pellerin et al., 2007; Suh et al., 2007). Moreover, studies in which computer models were used proposed that throughout high neuronal activity, an impediment of neuronal glycolysis is caused because of the inhibition of phosphofructokinase I, and as a consequence lactate from astrocytes becomes the favored neuronal energy substrate (Occhipinti et al., 2009).

### Glutamate-Glutamine Cycle

For years, it has been generally accepted that neurons release Glu that is taken up by astrocytes that convert it to Glutamine (Gln) through the action of the GS. Gln is then taken up by neurons where, by the action of the phosphate-activated glutaminase (PAG), it is converted into Glu that can be used to produce GABA through the action of the Glu decarboxylase (Figure 2) (Westergaard et al., 1995; Hertz et al., 1999; Calvetti and Somersalo, 2013).

Glu has different metabolic fates in astrocytes and neurons. For instance this neurotransmitter can either be converted to Gln or enter to the tricarboxylic acid cycle (TCAC) from which the cell may obtain lactate from malate (Figure 2) (Westergaard et al., 1995; McKenna, 2007). Glu/Gln metabolism is regulated through different mechanisms that include the availability and distribution of metabolite transporters and the quantity and activity of different key enzymes (McKenna, 2007). However the metabolic fate of Glu in astrocytes is mainly dependent on its external milieu concentrations (Westergaard et al., 1995; McKenna et al., 1996; McKenna, 2007). At low Glu extracellular concentrations, this compound is converted to Gln that can be used by neurons to replenish their Glu or GABA pools (Farinelli and Nicklas, 1992), while at higher concentrations Glu enters to the TCAC for lactate and aspartate formation (Sonnewald et al., 1993; Westergaard et al., 1995; McKenna et al., 1996; McKenna, 2007) (Figure 2).

### Glutamate Uptake by Astrocytes

When Glu is released from a neuron into the synapse its action as a neurotransmitter is regulated via uptake systems located in neurons and astrocytes (Drejer et al., 1982; Pellerin and Magistretti, 1994; Hertz et al., 1999). After Glu is released to the synaptic clefts, where it interacts with receptors in the postsynaptic neurons, Glu is taken up by highly efficient transporters present in surrounding cells, most of which are astrocytes (Anderson and Swanson, 2000; Šerý et al., 2015). Glu is mainly co-transported with three Na^+^ molecules into astrocytes, with the exit of a K^+^ or an OH^-^. Glu is also transported by a Na^+^ independent uptake mechanism which is generally chloride-dependent and it is executed by glutamate/cystine antiporters (Anderson and Swanson, 2000). Glu uptake by astrocytes is carried out through several transporters known as excitatory amino acid transporters (EAATs), among these EAAT1/GLAST and EAAT2/GLT1 are particularly located near glutamatergic synapses and reprensent the most abundant type of EAATs (Gegelashvili and Schousboe, 1998; Anderson and Swanson, 2000; Gadea and López-Colomé, 2001; Šerý et al., 2015). These receptors transport Glu against a high concentration gradient constantly taking Glu from the synapse. This constant uptake in glutamatergic neurons is necessary, both to finish the neurotransmitter effect that it produces in the synapse and to maintain low Glu levels to prevent excitotoxicity (Pellerin and Magistretti, 1994; Gadea and López-Colomé, 2001). Despite being constant, Glu uptake can be regulated, through modifications in transporter expression or by changes in these transporters activity that is closely related to ion grandients and membrane potential (Gegelashvili and Schousboe, 1998; Anderson and Swanson, 2000).

### Gluthation Related Redox Regulation

It is now common knowledge that the brain is the most vulnerable organ to oxidative damage (Dringen et al., 2000; Fernandez-Fernandez et al., 2012; Johnson et al., 2012; Maity-Kumar et al., 2015). It has been demonstrated that astrocytes play a key role in neuron antioxidant and energy metabolism, being gluthation (GSH) a pivotal player in astrocytic antioxidant defense (Fernandez-Fernandez et al., 2012; Dringen et al., 2000, 2014; Schreiner et al., 2015). GSH is known as the main cellular reductant and antioxidant compound. This tripeptide is present in high concentrations in cells and is essential in redox regulation and thiol pool regulation, besides playing a critical role in cell fate (Hiroi et al., 2005; Valko et al., 2007). GSH is metabolized in astrocytes, as well as other cell types, to Glu, Cysteine and Glycine through the action of the glutamine Cys ligase and the GSH synthetase (See Figure 2) (Dringen et al., 2000).

GSH levels are comparatively higher in astrocytes than neurons (Fernandez-Fernandez et al., 2012). Moreover, neurons present a relatively low expression of antioxidant enzymes and scavangers in general. Additionally, astrocytes are responsible for supplying neurons with the necessary substrates, especially Cysteine, to produce their own GSH (Sagara et al., 1993; Bolanos et al., 1996; Dringen et al., 1999, 2014). Thus, astrocytes become essential for ROS and reactive nitrogen species detoxification in neurons and for the maintenance of the metabolic and redox balance in these cells (Dringen, 2000; Fernandez-Fernandez et al., 2012; Narasimhan et al., 2012; Dringen et al., 2014).

## Metabolic Collaboration Between Neurons and Astrocytes Against Energy Failure During PA

Over the past 30 years the enormous range of functions astrocytes display, which result indispensable in order to maintain homeostasis in a healthy CNS, have been described. Given their fundamental role under physiological circumstances, the crucial contribution that they have in pathological mechanisms of severe diseases becomes evident. Astrocytes have developed several mechanisms that are crucial to overcome brain injury (Trendelenburg and Dirnagl, 2005).

When approaching the protective or destructive mechanisms manifested as a consequence of PA in the brain into the frame of astrocytic and neuronal interaction, many aspects must be taken into account. First of all, we consider that the target organ of study, the brain, is one of the most complex and multifaceted organs. We also have to consider the singular gestational and chronological age, and how it interacts with brain development. Furthermore, it is important to bear in mind the complexity of such an intricate stimulus as HI. This turns the task of experimentally trying to dissect neuroprotective mechanisms from the ones that result destructive very problematic, due to the fact that they both coexist and comprehend evolving processes.

### Metabolic Pathogenesis of PA

There is great agreement about the pathogenic mechanisms of PA. A massive body of evidence supports the neuropathology of PA as a general decrement in glucose and oxygen supply caused, most of the times, by hypoxia and ischemia during the perinatal period (Lai et al., 2011). Hypoxia is characterized by low oxygen supply into the tissues, especially the brain, and it is defined as the threshold in which oxygen concentrations limit normal cellular processes (Reyes et al., 2012). Ischemia, on the other hand, comprises a restraint in blood supply to different organs (Radulova and Slancheva, 2014). The brain injury as consequence of a PA involves an evolving process that, from a temporal point of view, is developed in two phases: the first one is initiated by a lack in oxygen and glucose supply that causes a primary energy failure (PEF), followed by a secondary energy failure (SEF) caused by reperfusion (Vannucci et al., 2004).

### Primary Energy Failure

The brain depends on oxygen to maintain aerobic metabolism, thus the reduction of oxygen supply makes the sustenance of aerobic metabolism, including mitochondrial oxidative metabolism, impossible. In fact, general mitochondrial metabolism was found to be reduced as far as 6 h after the HI insult (Morken et al., 2014a). As a consequence, oxidative phosphorylation becomes precluded and so does the electron transport chain, thus, a switch to anerobic metabolism takes place. The glycolysis pathway is activated, reflected in an increase in glucose transporters after HI, as a compensatory mechanism (Vannucci et al., 1998). At first, this is favorable for adaptation to oxygen deprivation, but eventually the consequences of this poor energy production pathway become evident since poor stores of glucose in brain tissue are unable to compensate the general discontinuation of glucose delivery taking place due to ischemia (Herrera-Marschitz et al., 2014; Morken et al., 2014a). In the context of a hypoxia, anaerobic glucose utilization derives in production of lactic acid (Hochachka and Mommsen, 1983; Siesjö, 1984). It is well known that lactate accumulation may cause acidosis, although it was found in rat pups that acidosis occurs independent of lactate concentrations (Vannucci, 1990; Welsh et al., 1990; Vannucci et al., 1992).

Although there is evidence showing that astrocytes, compared to neurons, are usually more resistant to ischemia and other damaging factors (Lukaszevicz et al., 2002), cultures of cortical astrocytes indicate that this type of cell is mainly vulnerable to acidosis (Giffard et al., 1990, 2000). Even though anaerobic glycolysis allows astrocyte survival, the increase of lactic acid throughout hypoxia has a negative impact in astrocyte metabolism. Under these circumstances, non-oxidative ATP production in cultured astrocytes cannot be maintained because the pH falls under 6.6 with serious and irreversible astrocytic damage (Swanson et al., 1997).

Long lasting episodes of mild acidosis (pH 6.8) affect astrocytes in culture, since they are more sensitive compared to neurons (Giffard et al., 1990). This could be attributed to the fact that astrocytes express a cotransporter of sodium bicarbonate which, during acidosis, mediates a sodium current into the astrocytes’ interior (Giffard et al., 2000).

Notwithstanding the evident detrimental consequences of lactate accumulation due to anaerobic metabolism, it has been shown that lactate serves as an alternative metabolic source for neurons. Moreover, it has been hypothesized that lactate may be the main substrate in the adult brain (Wyss et al., 2011) as well as aforesaid, in the developing CNS. This premise regarding lactate as the main source of energy for neurons is the foundation for the aforementioned “lactate shuttle hypothesis” (Pellerin et al., 2007). Moreover, in the context of ischemia, it has also been found that lactate represents an important alternate substrate to glucose (Young et al., 1991).

The effects of hypoxia imply a general depression of metabolism, but especially oxygen-dependent ATP production pathways, and thus, those pathways that depend on ATP consumption (Wu, 2002). This primary decrease in high energy phosphate levels provokes a failure in the ATP dependent Na^+^/K^+^ pump that leads to an intracellular accumulation of Na^+^, Ca^2+^ and water, leading to cytotoxic edema formation (Lai et al., 2011). These events favor membrane depolarization and an increased release of excitatory neurotransmitters, especially Glu. Thus, astrocytes and their interaction with neurons play an essential role in Glu clearance during excitotoxicity. Glu reuptake is done in a steep concentration gradient (Erecińska and Silver, 1990) and the amount of energy necessary to achieve this end requires a large amount of ATP consumption (Sibson et al., 1998a). As previously stated, acidosis affects non-oxidative ATP production in astrocytes, and thus it has consequences in carrying out normal Glu uptake, which is essential, particularly during HI, due to the exacerbated Glu release (Swanson et al., 1995). Indeed, astrocytic Glu uptake is essential for neuronal survival under neurotoxic circumstances. Vulnerability of neurons to Glu excitotoxicity is 100 times higher in absence of astrocyte intimate interaction (Rosenberg et al., 1989). Furthermore, an increase on glial transporters involved in Glu removal in pre-synapsis was observed in rats subjected to HI (Tao et al., 2001).

Excitotoxicity damage is also potentiated by various consequences of the energy failure: a disruption of the glutamine-glutamate cycle (Hagberg et al., 1987; Minc-Golomb et al., 1987; Choi et al., 1988; Danbolt, 2001) since ATP is the source of energy for glial glutamate transport (Sibson et al., 1998a), a net reduction in Glu reuptake by neurons from the synapsis after HI in rat hippocampus (Jabaudon et al., 2000), and finally Glu release from astrocytes, caused by elevation of extracellular K^+^ levels, as well as neurons Glu efflux (Rossi et al., 2000).

Since astrocytes, as well as neurons, fail to maintain Glu uptake the glutamine-glutamate cycle is disrupted and both types of cells release great amounts of the neurotransmitter, producing a subsequent receptor overstimulation. There is evidence that the immature brain is more susceptible to excitotoxicity than the adult brain, in fact, large injections of definir NMDA into rat brain structures result in more extensive cell loss in the neonate compared with the adult brain (McDonald et al., 1988). Moreover, a parallel between the sensitivity of the rat brain to the HI damage and NMDA toxicity has been found (Ikonomidou et al., 1989). This susceptibility might be associated to the fact that NMDA receptors not only are overexpressed, up to 2 times more, in the developing brain compared to the adult (McDonald et al., 1988; Tremblay et al., 1988), but also because they express a heterogenic functional pattern in the perinatal period that, given a excitatory stimuli, facilitates an extended and also prominent Ca^2+^ influx (Danysz and Parsons, 1998). Regarding Glu receptors, during the neonatal period they show greater density and function ability (McLean et al., 2004). It has also been evidenced that Glu receptors expresses peaks of affinity in the perinatal period of guinea pig fetus (Mishra et al., 1982).

This overstimulation of excitatory receptors leads to an increase in cytosolic Ca^2+^, and generation of ROS that results in an excito-oxidative injury cascade, including activation of phospholipase (Morken et al., 2014b). Many of these cascades converge into an ultimate target: the mitochondria (Hagberg et al., 2014) but ultimately lead to a massive and devastating cell loss. As we have previously stated astrocytes play a key role against a redox imbalance caused by a massive generation of ROS while their interaction with neurons is crucial for the survival of the latter during such an event (See “Gluthation Related Redox Regulation” section).

The features of the immature brain regarding an improvement of the excitatory system, potentiates excitotoxicity, exacerbating the damaging effects of the primary energy depletion.

PEF leads to an evolving and devastating collapse in the main cell functions that might provoke its very death or set the stage to SEF damage that will ultimately lead to massive cell loss.

### Secondary Energy Failure

When re-oxygenation occurs it is accompanied by its characteristic reperfusion injury, however without re-oxygenation survival would result impossible. SEF progress diverges across species and depends on the severity of the insult and its nature. There is certain agreement that there can be an early SEF between 8–16 h, or a late one (24–48 h after re-oxygenation) (Lorek et al., 1994; Penrice et al., 1997; Vannucci et al., 2004).

Glucose and oxygen deficiency that led to a primary cell death is followed by a secondary cell loss (Inder and Volpe, 2000) as a consequence of re-oxygenation. Oxidative metabolism in the mitochondria recommences and there is a transient recovery to baseline energy levels, observed from 2 to 3 hours after reperfusion. The micro-environment of the cells, already damaged by hypoxia, is now overflowed by oxygen that overwhelms mitochondrial oxidative phosphorylation capacity, which results in an accumulation of ROS (Ferreiro et al., 2001).

The mitochondria, while being a ROS massive producer, also have a particular susceptibility to ROS damage (Capani et al., 2001, 2003). These organelles are the primary target of a redox imbalance (Hagberg et al., 2014), ROS damage results in a long-lasting failure of this organelle that causes a second decline in energy levels observed across the following 48 h after the transient recovery (Lorek et al., 1994; Penrice et al., 1997; Vannucci et al., 2004; Romero et al., 2015). This secondary ATP depletion has been observed in both, human and animal studies based on superior index format for “31” magnetic resonance spectroscopy, that shows that after an early recovery interval, consisting in a phosphorus spectra restitution upon resuscitation from asphyxia, there is indeed a secondary decline in energy status constituted by tissue depletion of phosphocreatine, ATP or both compounds (Lorek et al., 1994; Penrice et al., 1997; Azzopardi et al., 2012). These studies have shown that the secondary failure in cerebral energy status after HI is a significant contributor to the ultimate brain damage (Williams et al., 1992). Moreover, survival duration of the animals under anoxic circumstances in 7 day old rats shows an inverse correlation with cerebral metabolic rates (Duffy et al., 1975). In spite of these results, studies conducted by Vannucci et al. (2004) indicate that SEF following HI is not the cause, but a consequence of ultimate brain damage (Towfighi et al., 2004).

Furthermore, it has been postulated that the cause of mitochondrial metabolic impairment may not be the lack of oxygen *per se*, but other detrimental factors related to the excito-oxidative cascade (Niatsetskaya et al., 2012; Hagberg et al., 2014). Indeed, glutamatergic excitotoxicity ultimately leads to neuronal death (Choi et al., 1988), being one of the major causes of cellular loss as a result of neonatal HI (Johnston et al., 2011). The aforementioned hypothesis was also confirmed by Morken et al. (2014a) in a rat model of neonatal HI in which they observed a decrease in mitochondrial metabolism in neurons, long after complete reperfusion and the reestablishment of oxygen delivery. Moreover, novel data from our laboratory supported the idea of general alterations of the redox state. Overexpression of thioredoxins 1 and glutaredoxins 2, which are two major members of the thioredoxin family in SH-SY5Y line culture subjected to hypoxia-reoxygenation showed to contribute to neuronal integrity (Romero et al., 2015).

Taking these results into consideration, it seems fair to conclude that there is a direct association between SEF and delayed, irreversible metabolic crisis, whereas it remains unclear if there is a parallelism between temporal evolution and cell death and that these events are strongly tied to neuron and astrocytes as well as their interactions.

## Conclusion

To our knowledge, the role of neuron-astrocyte interaction in relation to PA has never been revised to the moment. Here we have summarized many of the aspects that we think should be taken into account when studying PA, along with the newest advances in the field.

Given that the perinatal period is of particular importance for the developing brain, taking into account the continuous collaboration between astrocytes and neurons seems to be essential when studying PA pathogenic mechanisms and effects in the developing brain. As aforesaid, the intricate relationship between these cell types plays a decisive role in the normal functioning of the developing CNS. Besides the crucial part they both play under healthy circumstances, there have been also great advances in identifying how this interaction works when brain insult occurs, influencing not only the development of brain injury in the perinatal period, but also the recovery. We have extensively revised several advantages that each cell type has, compensating the disadvantages of the other one, the molecular and signaling pathways that could be activated under HI conditions and that enable crucial collaboration during energy failure.

The aforementioned aspects in this work should be taken into consideration in the development of future treatments since most of the findings point towards astrocyte-neuron metabolic interaction as potential therapeutic targets. These findings should also stimulate further research to resolve and confirm some of the discussions regarding topics such as the actual contributions of SEF to ultimate cell loss, the critical role of lactate and other substrates as alternative metabolic sources or as decisive contributors to detrimental acidosis under PA, and the role of mitochondrial dysfunction and its damaging consequences over astrocyte normal functioning, especially over glutamate-glutamine cycle that ultimate affects neuron as well.

## Author Contributions

All authors contributed in some way to the writting process of this review. All authors listed, have made substantial, direct and intellectual contribution to the work, and approved it for publication.

## Conflict of Interest Statement

The authors declare that the research was conducted in the absence of any commercial or financial relationships that could be construed as a potential conflict of interest.
